# Fluvalinate-Induced Changes in MicroRNA Expression Profile of *Apis mellifera ligustica* Brain Tissue

**DOI:** 10.3389/fgene.2022.855987

**Published:** 2022-04-12

**Authors:** Chao Tianle, Yang Liuxu, Lou Delong, Fan Yunhan, He Yu, Shan Xueqing, Xia Haitao, Wang Guizhi

**Affiliations:** ^1^ Shandong Provincial Key Laboratory of Animal Biotechnology and Disease Control and Prevention, College of Animal Science and Veterinary Medicine, Shandong Agricultural University, Tai’an, China; ^2^ Comprehensive Testing and Inspection Center, Shandong Provincial Animal Husbandry and Veterinary Bureau, Jinan, China; ^3^ Animal Husbandry Development Center of Linqu County, Weifang, China

**Keywords:** *Apis mellifera ligustica*, fluvalinate, brain, transcriptome, MicroRNA

## Abstract

Fluvalinate is a widely used and relatively safe acaricide for honeybees, but it still has a negative impact on honeybee colonies. Such negative effects may be related to fluvalinate-induced brain nerve tissue damage, but the detailed molecular regulatory mechanism of this phenomenon is still poorly understood. In this study, we analyzed the miRNA expression profile changes in the brain tissue of *Apis mellifera ligustica* by miRNA sequencing after fluvalinate treatment. A total of 1,350 miRNAs were expressed in *Apis mellifera ligustica* brain tissue, of which only 180 were previously known miRNAs in honeybees. Among all known and novel miRNAs, 15 were differentially expressed between at least two of the four time periods before and after fluvalinate administration. Further analysis revealed five significantly enriched KEGG pathways of the differentially expressed miRNA (DEM) potential target genes, namely, “Hippo signaling pathway-fly,” “Phototransduction-fly,” “Apoptosis-fly,” “Wnt signaling pathway,” and “Dorso-ventral axis formation,” which indicates that differentially expressed miRNA function may be related to cell apoptosis and memory impairment in the fluvalinate-treated *Apis mellifera ligustica* brain. Ame-miR-3477-5p, ame-miR-375-3p, and miR-281-x were identified as key miRNAs. Overall, our research provides new insights into the roles of miRNAs in brain tissue during the process of fluvalinate-induced *Apis mellifera ligustica* poisoning.

## Introduction

Among all of the commonly used anti-mite insecticides for honeybees, fluvalinate is known to have low toxicity (Johnson et al., 2006; Johnson et al., 2009). However, there is growing evidence that fluvalinate is not entirely benign for *Apis mellifera*. It has been reported that honeybee workers that received field-level fluvalinate treatment showed a higher mortality rate than those that did not receive treatment (Medici et al., 2012; Berry et al., 2013). *Apis mellifera* individuals exposed to fluvalinate have increased mortality, lower body weight, reduced reproductive viability, and decreased learning and memory ability (Rinderer et al., 1999; Burley et al., 2008; Berry et al., 2013; Frost et al., 2013). Furthermore, as the primary molecular target for pyrethroid insecticides, the *Apis mellifera* voltage-dependent sodium channel has been reported to have a higher affinity than a variant of the *Varroa destructor* voltage-dependent sodium channel for fluvalinate (Soderlund, 2010; Gosselin‐Badaroudine and Chahine, 2017). Although there is no direct evidence to date, growing evidence of memory loss, learning ability decrease, and toxicity for voltage-gated sodium channels indicate that the lethal and sublethal effects of fluvalinate on honeybees may be partly due to nervous system damage.

MicroRNA is a type of nonprotein-coding endogenous small RNA molecule with a sequence length of 20–24 nt. By inhibiting or degrading mRNA, it is involved in many biological regulatory processes at the posttranscriptional level in plants, animals, and microorganisms and it participates in many pathways *in vivo*, such as regulating cell proliferation, differentiation, apoptosis, stress response, and metabolism (Griffiths-Jones 2004; Calin and Croce, 2006). Some effects of miRNAs on the nervous system of *A. mellifera ligustica* have been reported.

This study used transcriptome sequencing technology to reveal changes in the miRNA expression profiles of *Apis mellifera ligustica* brain tissue under the influence of fluvalinate, identify miRNAs that were differentially expressed before and during fluvalinate treatment, and study the potential function of these miRNAs at different time points after fluvalinate treatment. The miRNAs involved in the fluvalinate response were revealed, and functional enrichment analysis of the miRNA target genes was used to perform a preliminary assessment of the pathways and mechanisms by which miRNAs are involved in regulating this process. Our study provides new insights into miRNA function in fluvalinate-induced honeybee brain tissue molecular regulation changes.

## Materials and Methods

### Sample Collection

We took six colonies of *Apis mellifera ligustica* with similar colony potential and randomly divided them into two sets (six colonies in each set). Each colony lives in a standard sixteen-frame hive with one naturally mated queen inside, and the colony size was similar between the two sets (approximately 16,000–18,000 workers in each colony). One was set as the control and the other as the test group. The test groups were exposed to fluvalinate (fluvalinate strip, Sichuan Pengshan Wangshi Animal Health Co., Ltd.) at a normal dosage for administration to honeybees (according to the instructions of the drug, a single strip was placed in the beehive every 7 days, which contained 40 mg of fluvalinate). For all of the colonies, we sampled foragers that returned from 08:00 to 12:00. Sample honeybees were taken 1 day before and 10, 20, and 30 days after fluvalinate treatment began, and 20 adult worker honeybees were collected from each bee colony for each sampling time. All collected honeybees were immediately used for dissection under a microscope, and 20 bee brains collected from the same colony were pooled for subsequent RNA extraction. The royal jelly glands and pigments were removed from the brain, and the brain tissue was immediately stored in an RNA preservation solution (Beijing Tiangen Biochemical Technology Company). The sampling site was Fuxin Honeybee Farm in Tai’an City, Shandong Province, China, which provided a uniform breeding environment for the experimental animals.

### RNA Extraction, Library Construction, and Sequencing

We used TRIzol to extract total RNA from each sample, and the RNA molecules in a size range of 18–30 nt were then enriched by polyacrylamide gel electrophoresis (PAGE). The RNA quality was evaluated with an Agilent 2100 Bioanalyzer. The total RNA samples satisfied the required quality of RNA integrity number (RIN) > 7.0. Then, 1 μg RNA per sample was used for library construction according to the Illumina TruSeq sample RNA Sample Prep Kit (Cat#RS-200-0012). Sequencing was performed by Guangzhou Gene Denovo Biotechnology Co., Ltd., using an Illumina HiSeq TM 2500. In particular, libraries from untreated brain tissue samples were named CK1, CK2, and CK3; libraries from samples collected 10 days after fluvalinate treatment were named D10-1, D10-2, and D10-3; libraries from samples collected 20 days after fluvalinate treatment were named D20-1, D20-2, and D20-3; and libraries from samples collected 30 days after fluvalinate treatment were named D30-1, D30-2, and D30-3.

### Sequencing Data Quality Control

The low-quality reads were filtered out of the data to obtain high-quality reads. Then, reads without 3′ linkers and 5′ linkers, reads without inserts or with inserts less than 18 nt in length, and polyA reads were removed. Next, small RNA tags with a read frequency lower than two were also removed. Finally, we obtained clean small RNA tag sequences.

### Sequencing Data Alignment

The software Blastall 2.2.25 (blastn) was used to align all the clean small RNA tags with the small RNAs in GenBank and compare them with the small RNAs in the Rfam database (release 11.0) (Griffiths-Jones et al., 2003) to identify and remove rRNA, scRNA, snoRNA, snRNA, and tRNA. Bowtie software (version 1.1.2) was used to compare all clean reads with the *A. mellifera ligustic*a reference genome (GCF-003254395.2), and all the reads mapped to exons, introns, and repeat sequences were removed. According to the genome alignment results, the small RNA tag sequence set that characterized each group was determined.

We also used Bowtie (version 1.1.2) alignment to identify the known miRNAs of *A. mellifera ligustica*. The same method was used to compare the miRNA sequences of all the species in the miRbase database (V22) (Griffiths-Jones et al., 2006) to identify homologous miRNAs. Then, the clean reads that had not yet been annotated were compared with the reference genome, and novel miRNAs were predicted based on the genome position and hairpin structure predicted by MIREAP V0.2 software (Wang et al., 2015).

### Identification of Differentially Expressed MicroRNA

The miRNA expression abundance was evaluated by the transcripts per million reads (TPM). The edgeR package was used to perform a differential expression analysis of the miRNAs, and miRNAs with |log2(FC)| > 1 and FDR < 0.05 were identified as DEMs.

### MicroRNA Target Gene Prediction

RNAhybrid (version 2.1.2) (Krüger and Rehmsmeier, 2006) + svm-light (version 6.01) (Joachims, 1999), Miranda (version 3.3a), and TargetScan (version 7.0) (Turner, 1985; Lewis et al., 2005) were applied for miRNA target gene prediction. The intersection of the software results was taken as the potential miRNA target gene set.

### Differentially Expressed MicroRNA Expression Profile Clustering

The z-score of each DEM was calculated using a tpm value. Using the MultiExperiment Viewer (MeV) (Saeed et al., 2003), hierarchical clustering was performed with the z-score.

### Quantitative Real-Time PCR (qRT-PCR) Validation of Differentially Expressed MicroRNA

To further verify the accuracy of our sequencing data, 10 DEMs were selected for the qRT-PCR test. A LightCycler 480 real-time system (Roche, United States) was applied for the experiment. The detailed information of primers for qRT-PCR testing was listed in Supplementary Table S1, and U5 snRNA and 18S rRNA (Cristino et al., 2014; Mello et al., 2014) were selected as the reference gene. qRT-PCR was performed according to the instructions of the Mir-X™ miRNA qRT-PCR TB Green™ Kit (Clontech). All reactions were performed using three biological replicates, and each biological replicate had three technical replicates. The qRT-PCR testing results were calculated using 2^−ΔΔCT^ ± SEM, in which the geometric mean of the threshold cycles (Ct) values of the two reference genes was used for the calculation, and the significance of the difference in miRNA expression was tested using Tukey’s honestly significant difference (HSD).

### Functional Enrichment Analysis

In order to generate a comprehensive description of the biological characteristics of the DEMs, potential target genes of DEMs from three different expression profile clusters were respectively enriched into the Gene Ontology (GO) database (Ashburner et al., 2000) and Kyoto Encyclopedia of Genes and Genomes (KEGG) (Kanehisa et al., 2008) with FDR ≤ 0.05 using the R package clusterProfiler (Yu et al., 2012).

### Network Analysis

We used Cytoscape 3.7.0 to construct the miRNA-KEGG pathway network diagram. Based on the results of KEGG enrichment, we used the significantly enriched KEGG pathways and 15 DEMs as nodes to make a network diagram. DEM node sizes were determined by the expression values.

## Results

### Assessment of Sequencing Quality

A total of 184,051,902 clean reads were obtained by sequencing. The sample with the most clean reads was D30-3, with 18,694,375 clean reads, and the lowest was CK-1, with 9,844,859. The number of clean tag sequences in each sample ranged from 18,109,705 to 9,626,768, and the filtering rate ranged from 94% to 98%. The number of reads in each sample library was within a reasonable range, and there were no abnormal samples (Supplementary Table S2).

### Alignment Analysis

Comparisons were used to determine the miRNA contents present in the samples and their base distributions and identify known and novel miRNAs. The proportion of previously known miRNAs, that is, the honeybee miRNAs included in miRBase, was above 80%. The proportions of the miRNAs known from other species but not annotated in the honeybee miRBase data were all approximately 2%, while some newly discovered miRNAs accounted for only 0.1%. Overall, 1,350 miRNAs were detected, of which only 180 were previously known miRNAs in honeybees, while 924 were known by comparison with other species’ known miRNAs, and 246 were newly discovered novel miRNAs (Supplementary Table S3).

### Analysis of Differential MicroRNA Expression

**FIGURE 1:**
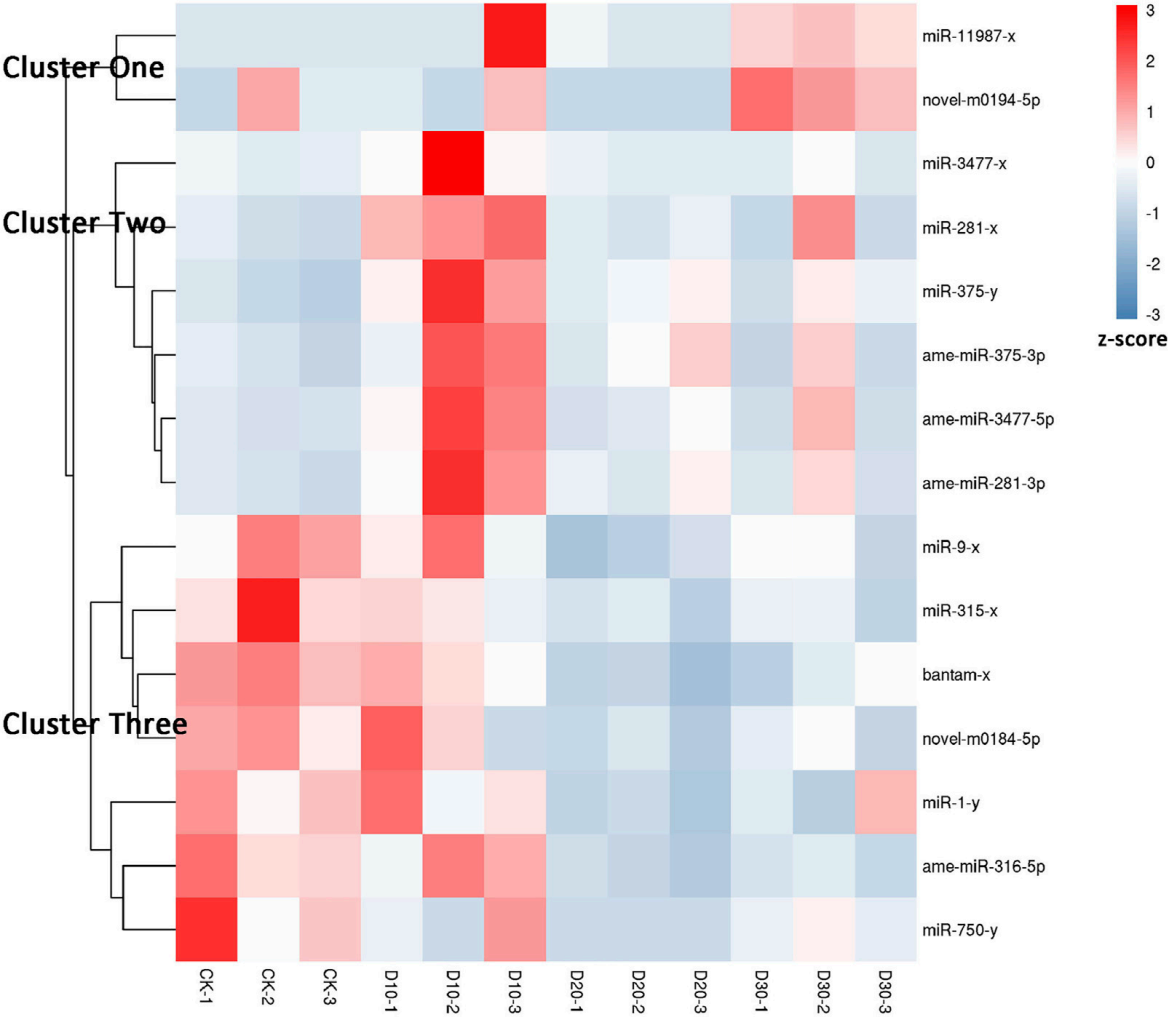
Expression pattern cluster analysis of differential expressed miRNAs. Each column represents a sample, and each row represents a miRNA. The expression level of miRNA in different samples is indicated by different colors. The redder the color, the higher the expression level, and the bluer the color, the lower the expression level.

**FIGURE 2:**
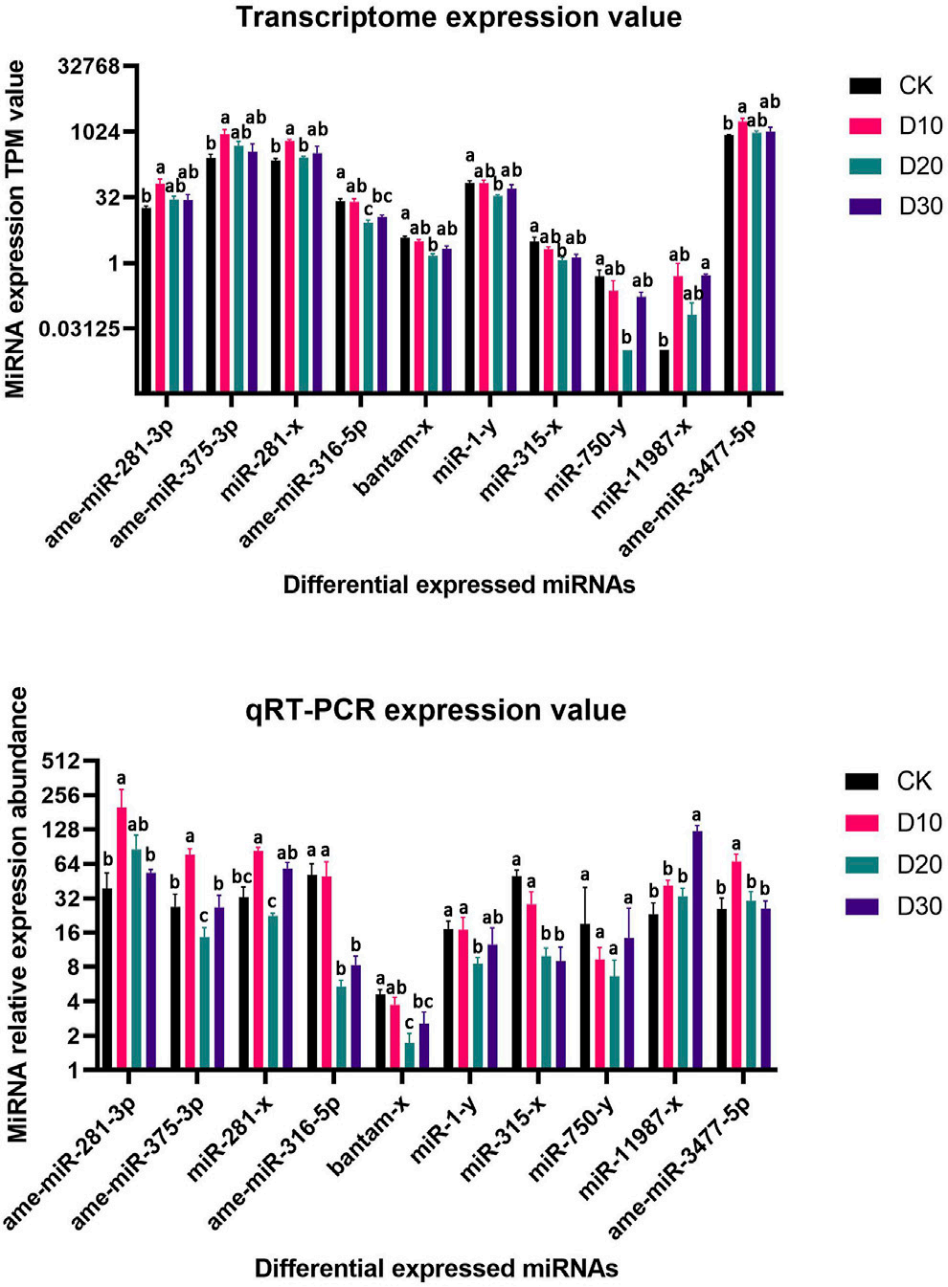
RNA-Seq and qRT-PCR relative expression level of differential expressed miRNAs. **(A)** MiRNA expression TPM value of RNA-Seq, **(B)** MiRNA relative expression level of qRT-PCR. Significant differences are indicated by different symbols with FDR < 0.05 in RNA-Seq results and *p* < 0.05 in qRT-PCR results.

Using |log2(FC)| > 1 and FDR < 0.05 as the thresholds, 15 miRNAs were identified as differentially expressed among at least two different time points before or after fluvalinate exposure (Supplementary Table S4). A heatmap of the expression levels of these 15 DEMs in each group was drawn, and expression profile cluster analysis was performed (Figure 1). All 15 DEMs were divided into three clusters, and DEMs included in each cluster had similar expression patterns. Among them, 10 DEMs were selected for qRT-PCR testing to validate the accuracy of the sequencing data. As shown in Figure 2, the qRT-PCR results of 9 miRNAs were consistent with the RNA-seq results. The miR-750-y qRT-PCR results did not show a trend of differences among the different time periods.

Two DEMs were contained in Cluster one, and their expression levels showed a continuous upward trend with increasing treatment time. In Cluster two, six DEMs showed the highest expression in the D10 stage. In Cluster three, the expression of the seven DEMs gradually decreased after treatment with fluvalinate. A reasonable guess is that miRNAs with the same expression pattern have similar functions. Therefore, to further explore the regulatory roles of these DEMs, we performed GO and KEGG enrichment analyses on the target genes of the three clusters of DEMs.

### Gene Ontology Enrichment Analysis

To clarify the main functions and regulatory mechanisms associated with the DEMs, we performed GO analysis on their potential target genes (Supplementary Figure S1).

For Cluster one target genes, 40 biological process terms and six molecular function terms showed significant enrichment, while no significant result was obtained from the cellular component terms. Among all Cluster one enriched GO terms, the top 12 most significant terms were all related to RNA splicing and processing.

For Cluster two, 61 cellular component terms, 54 molecular function terms, and 71 biological process terms were significantly enriched. It is worth noting that, among the three types of terms, the terms with the highest significant enrichment level were all related to ion transmembrane transport and protein modification.

For Cluster three, 10 cellular component terms, 43 molecular function terms, and 44 biological process terms achieved significant enrichment levels. The top significantly enriched biological process terms were “cellular protein modification process, protein modification process, and macromolecule modification.”

Furthermore, we also noticed that 27 GO terms were significantly enriched in all three clusters. Among them, we found three biological processes related to advanced brain functions: “behavior,” “learning or memory,” and “cognition.”

### Kyoto Encyclopedia of Genes and Genomes Enrichment Analysis

We performed KEGG pathway enrichment analysis on the target genes of the DEMs. The pathways that obtained significant enrichment are shown in Supplementary Table S5.

The results showed that, in Cluster one, 200 target genes were enriched in 53 KEGG pathways, and only five pathways were significantly enriched. Among them, the pathway with the most significant enrichment level and the largest number of target genes was “Dorsoventral axis formation.”

For Cluster two, 89 KEGG pathways were enriched, and 15 pathways achieved a significant level. Among them, the pathway with the most significant enrichment and the largest number of target genes was the “Wnt signaling pathway.”

In Cluster three, 749 target genes were enriched in 105 KEGG pathways, 11 of which were significantly enriched. The pathway with the most significant enrichment was “Phototransduction-fly,” and the pathway containing the largest number of target genes was “Wnt signaling pathway.”

**FIGURE 3:**
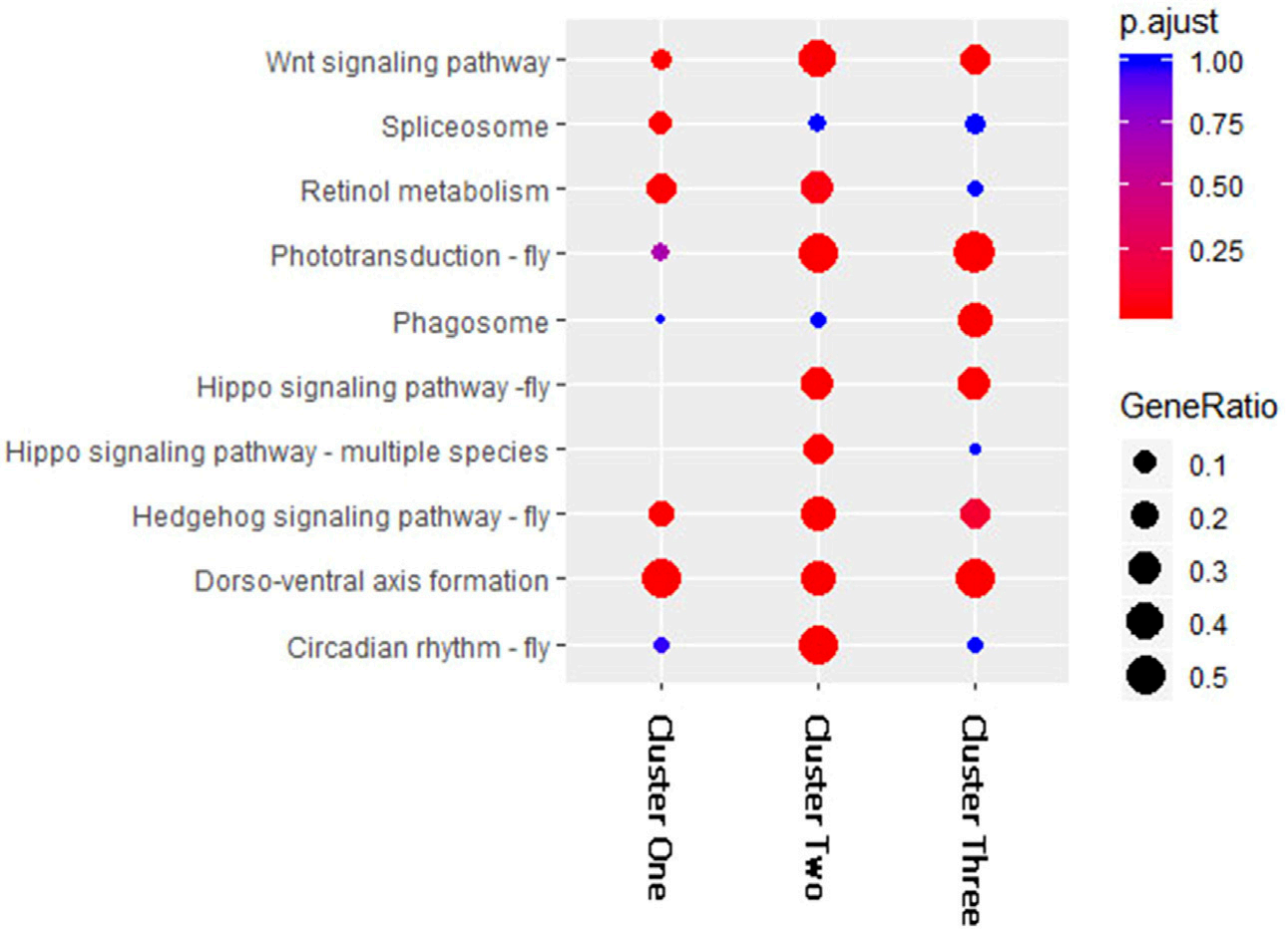
KEGG enrichment for miRNA target genes of each cluster. The horizontal axis of the bubble graph represents the number of target genes in each KEGG pathway, the vertical axis represents different channel names, the different colors of bubbles represent the FDR values, and the size of the bubbles represents the gene ratio value.

To compare the KEGG pathway enrichment results of the three clusters of DEMs, we selected and combined the top five enriched pathways in each cluster (Figure 3). The results showed large differences among the three clusters. The KEGG pathways “Dorso-ventral axis formation,” “Wnt signaling pathway,” and “Hedgehog signaling pathway-fly” were significantly enriched in all three DEM clusters, which may indicate that these pathways are regulated by DEMs at multiple periods after fluvalinate treatment. The pathways “Phototransduction-fly” and “Hippo signaling pathway-fly” were significantly enriched in Clusters two and three, which may indicate that these pathways are regulated by DEMs immediately after fluvalinate treatment. The pathways “spliceosome,” “circadian rhythm-fly,” and “phagosome” were specifically significantly enriched in Clusters one, two, and three, respectively, which may indicate that these pathways are related to the unique functions of DEMs with different expression patterns.

**FIGURE 4:**
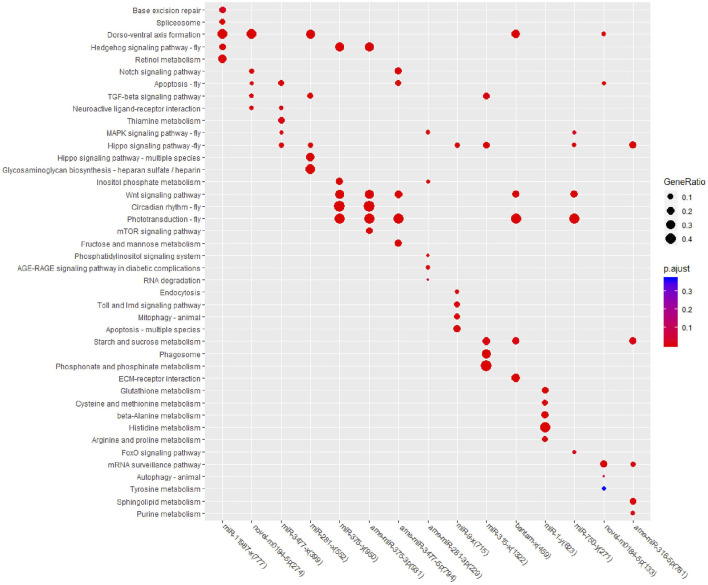
KEGG enrichment for target genes of each miRNA.

To further confirm the pathways that each miRNA may regulate, KEGG enrichment analysis was also performed for all 15 DEM target genes, and the top five enriched pathways were selected for comparison (Figure 4). In Cluster one, target genes were enriched in nine KEGG pathways, and only “Dorso-ventral axis formation” achieved the top five significance levels in both DEMs. In Cluster two, target genes were enriched in 20 pathways, while the pathways “Wnt signaling pathway” and “Phototransduction-fly” contained the most target genes and DEMs. In Cluster three, target genes were enriched in 23 pathways, among which target genes of four DEMs were enriched in “Hippo signaling pathway-fly,” and target genes of three DEMs were enriched in “Phototransduction-fly” and “Starch and sucrose metabolism.” Among all of the top enriched pathways, the “Hippo signaling pathway-fly” achieved the top five significant levels in six DEMs with the largest DEM number, followed by the “Dorso-ventral axis formation,” “Wnt signaling pathway,” and “Phototransduction-fly” pathways; all achieved the top five significant levels in five DEMs. According to the above enrichment results, “Hippo signaling pathway-fly,” “Dorso-ventral axis formation,” “Wnt signaling pathway,” and “Phototransduction-fly” were considered the key KEGG pathways in the miRNA coordinated posttranscriptional regulation after fluvalinate treatment.

### Network Analysis

**FIGURE 5:**
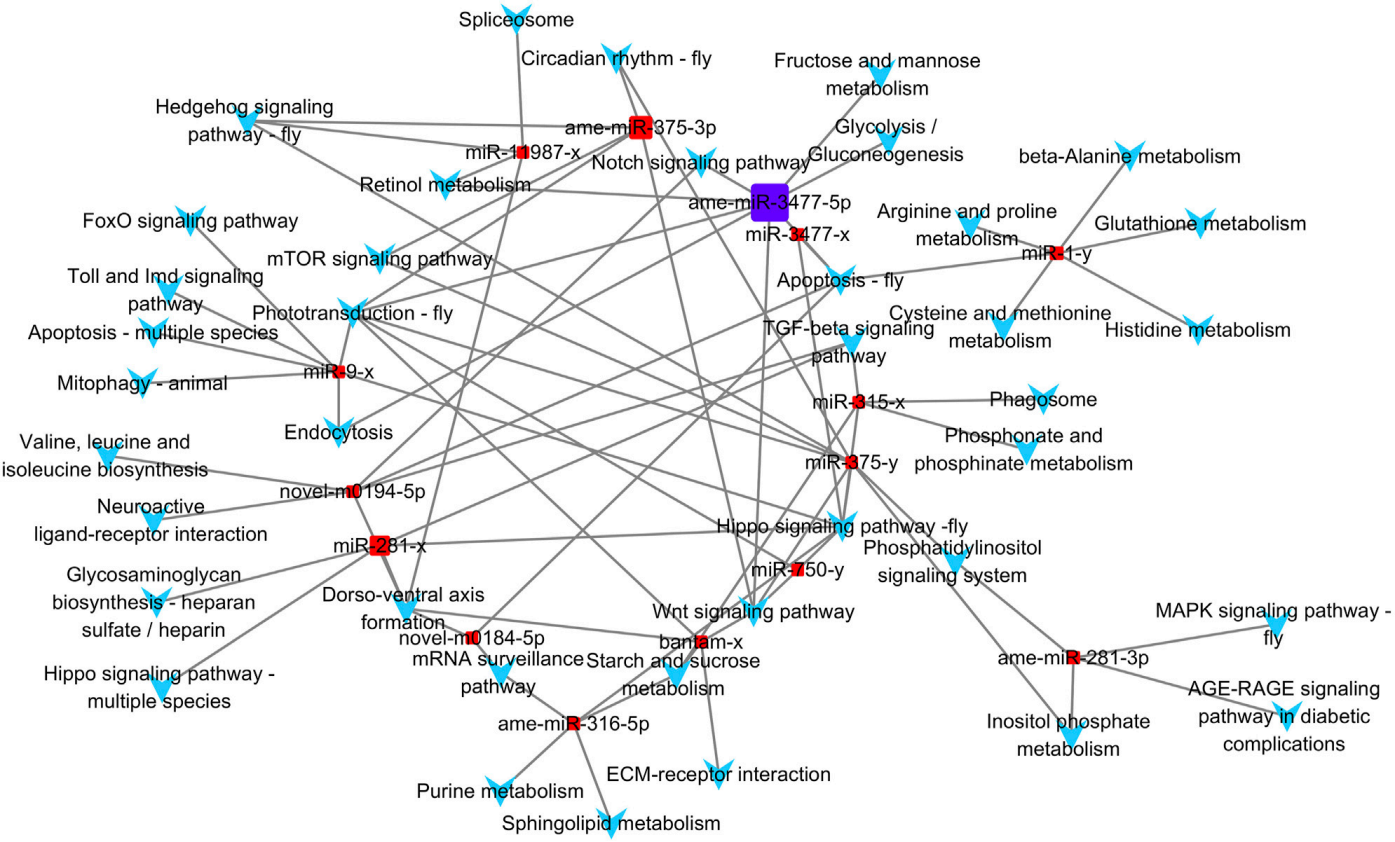
Differential expressed miRNA and KEGG pathway network. Square nodes represent miRNAs, and arrow-shaped nodes represent pathways. The miRNA nodes' sizes were determined by the expression value.

Based on the KEGG enrichment results, we imported the DEMs and all KEGG pathways with significant enrichment as nodes into Cytoscape 3.7.0 to draw a network map, and the size of the miRNA nodes was determined by their expression level. In total, 15 miRNAs and 39 significantly enriched KEGG pathways were included in the network (Figure 5). The most highly expressed miRNA was ame-miR-3477-5p, which exceeded the sum of the other 14 miRNAs, and its potential target genes were significantly associated with eight pathways.

Among the 39 significantly enriched KEGG pathways, “Hippo signaling pathway-fly” was associated with the target genes of seven miRNAs. Both the “Phototransduction-fly” and “Apoptosis-fly” pathways were associated with the target genes of six miRNAs, while the “Wnt signaling pathway” and “Dorso-ventral axis formation” were associated with the target genes of five miRNAs. Therefore, “Apoptosis-fly” was also considered a key KEGG pathway, similar to the other four pathways. DEMs with the top three expression levels that participate in the regulation of at least one key pathway were determined to be key miRNAs. Therefore, ame-miR-3477-5p, ame-miR-375-3p, and miR-281-x were identified as key miRNAs, and their target genes enriched in key KEGG pathways are listed as potential key target genes (Supplementary Table S6).

## Discussion

This study focused on identifying *Apis mellifera ligustica* brain tissue DEMs before and after fluvalinate treatment. As a pyrethroid, fluvalinate has been used to control Varroa mites in honeybees since the 1980s (Vu et al., 2020). In recent years, beekeepers have noted an increase in certain phenomena: honeybees gradually die and homing decreases after fluvalinate use, colonies generally decline, and empty nests often appear. Many studies have reported that fluvalinate has negative impacts on multiple survival traits of *Apis mellifera*, including learning and memory ability (Rinderer et al., 1999; Burley et al., 2008; Berry et al., 2013; Frost et al., 2013). However, to our knowledge, there is still no research on the function of miRNAs in this process.

In this study, 15 DEMs were identified at different times after fluvalinate exposure in *Apis mellifera ligustica* brain tissues. Among them, the five DEMs with the highest expression were ame-miR-3477-5p, ame-miR-375-3p, miR-281-x, miR-1-y, and ame-miR-281-3p. Ame-miR-3477-5p showed the highest expression value, which exceeded the sum of the other 14 miRNAs.

Expression pattern analysis showed that ame-miR-3477-5p reached the peak expression level at 10 days after fluvalinate treatment and then returned to the preadministration level. This phenomenon may suggest that ame-miR-3477-5p plays a role in the early brain damage or detoxification process caused by fluvalinate. However, we also found very few research reports related to ame-miR-3477-5p, and it has only been detected in three species: *Apis mellifera*, *Tribolium castaneum*, and *Nasonia vitripennis*. The only research related to ame-miR-3477-5p′s function reported that after worker honeybees perform visual pattern learning in a maze, the expression of miR-3477 in the brain is significantly upregulated (Qin et al., 2014). In our research, the potential target genes of ame-miR-3477-5p were significantly enriched in eight KEGG pathways, while the top enriched pathway was phototransduction-fly. This result suggests that the function of ame-miR-3477-5p may be related to vision, learning, or memory.

DEMs ame-miR-375-3p and miR-281-x, which were also identified as key miRNAs, showed similar expression patterns to ame-miR-3477-5p. Among them, the target genes involved in the key KEGG pathways “Phototransduction-fly” and “Wnt signaling pathway” of ame-miR-375-3p were highly similar to those of ame-miR-3477-5p. Both ame-miR-3477-5p and ame-miR-375-3p regulate the phototransduction pathway and Wnt signaling pathway mainly by targeting CaMKII (both target 42 transcripts of CaMKII). CaMKII is a multifunctional serine/threonine protein kinase that is highly expressed in brain tissue; it is also the main component of PSD and is autophosphorylated to participate in learning, memory, and cognitive ability and may be the molecular basis of nervous system plasticity (Joiner and Griffith, 1997; Bejar et al., 2002; Lisman et al., 2002; Zhang et al., 2005). Previous studies have confirmed that CaMKII is a molecular memory switch in vertebrates and insects and plays important roles in memory formation in *Drosophila* and *Apis mellifera* (Mehren and Griffith, 2004; Malik et al., 2013; Matsumoto et al., 2014; Scholl et al., 2015). Furthermore, activation of CaMKII was reported to be involved in the modulation of the voltage dependence of steady-state inactivation, and activation of the CaM-kinase II pathway by an increased intracellular calcium concentration might cause specific ion channels to be sensitive to the pesticide (Lavialle-Defaix et al., 2010). Several miRNAs, including miR-1, miR-30b-5p, miR-145, miR-148, and miR-152, have been reported to target the CaMKII gene, but miR-375-3p and miR-3477-5p are not among them (Terentyev et al., 2009; Liu et al., 2010; Cha et al., 2013; He et al., 2013). Our results reveal that ame-miR-375-3p and ame-miR-3477-5p are newly discovered potential regulators of CaMKII and may play roles in memory or cognitive impairment in the *Apis mellifera ligustica* brain after fluvalinate treatment. It is worth noting that the monkey DEMs bantam-x and miR-750-y were also predicted to target CaMKII, which may indicate the existence of a complex posttranscriptional regulatory network controlling CaMKII.

The key miRNA miR-281-x, which is also known as ame-miR-281-5p, has been reported to be differentially expressed in the honeybee brain after treatment with dinotefuran (Huang et al., 2021). In this research, the potential target genes of miR-281-x were significantly enriched in the key pathways Dorso-ventral axis formation and Hippo signaling pathway-fly. Our results showed that miR-281-x targets 27 transcripts of the LOC409780 gene, currently referred to as CELF2 (CUGBP Elav-like family member 2) in *Apis mellifera*. As a member of the CELF family and the dorsoventral axis formation pathway, CELF2 is widely expressed in various systems of animals and is implicated in the regulation of RNA biogenesis as a splicing factor (Dasgupta and Ladd, 2012; Ajith et al., 2016). MiR-281-x also targets 13 transcripts of the LOC408616 gene, currently known as TEAD1/TEF-1 (transcriptional enhancer factor TEF-1) in *Apis mellifera*. As a Hippo signaling effector, TEAD1 participates in various biological processes and plays important roles in animal tissue growth and maintenance (Dey et al., 2020; Heng et al., 2021).

The current results also show that the potential target genes of DEMs miR-11987-x, novel-m0194-5p, and novel-m0184-5p are enriched in the dorsoventral axis formation pathway, and both can target the Broad-Complex (Br-c) gene. As a member of the “Dorso-ventral axis formation” pathway, Br-C is an early ecdysone-responsive gene encoding a family of zinc-finger transcription factors and is involved in insect development regulation (Paul et al., 2006; Jiang et al., 2015). Br-C was reported to be relatively highly expressed in the brain tissue of honeybee and oriental river prawn, which may indicate that Br-C could participate in insect brain function regulation (Paul et al., 2006; Jiang et al., 2015). Interestingly, our results showed that DEMs miR-11987-x, novel-m0194-5p, and novel-m0184-5p have different target sites in the Br-C gene. miR-11987-x can target two transcripts, while novel-m0194-5p and novel-m0184-5p target seven different transcripts of Br-C.

In summary, the results of this study reveal that the miRNA expression profiles in the brains of *A. mellifera ligustica* changed before and after treatment with fluvalinate. The results of target gene functional enrichment analyses indicate that the DEMs may be related to cell apoptosis and memory damage in the brain. This study further confirms that abnormal activities observed among honeybees after fluvalinate administration may be caused by neurological disorders, including excessive arousal and memory loss caused by fluvalinate-induced brain damage. However, the specific roles of these miRNAs in brain function remain to be investigated, and their regulatory mechanisms also need to be further studied. This study provides new information on the molecular mechanisms and regulatory pathways of the fluvalinate response in *A. mellifera ligustica* brain tissue.

In summary, the results of this study reveal that the miRNA expression profiles in the brains of *A. mellifera ligustica* changed before and after treatment with fluvalinate. The results of target gene functional enrichment analyses indicate that DEMs may be related to cell apoptosis and memory damage in the brain. This study further confirms that abnormal activities in honeybees after fluvalinate administration may be caused by neurological disorders, including excessive arousal and memory loss caused by fluvalinate-induced brain damage. However, specific roles of these miRNAs in brain function remain to be investigated, and their regulatory mechanisms also need to be further studied. This study provides new information on the molecular mechanisms and regulatory pathways of fluvalinate response in *A. mellifera ligustica* brain tissue.

## Data Availability

The datasets presented in this study can be found in online repositories. The names of the repository/repositories and accession number(s) can be found below: https://www.ncbi.nlm.nih.gov/geo/, GSE162423.
