# Supplementary figures and images for: Fluvalinate-Induced Changes in MicroRNA Expression Profile of Apis mellifera ligustica Brain Tissue
Source: Front Genet. 2022 Apr 12;13:855987. doi: 10.3389/fgene.2022.855987 (PMC9039055; doi:10.3389/fgene.2022.855987)

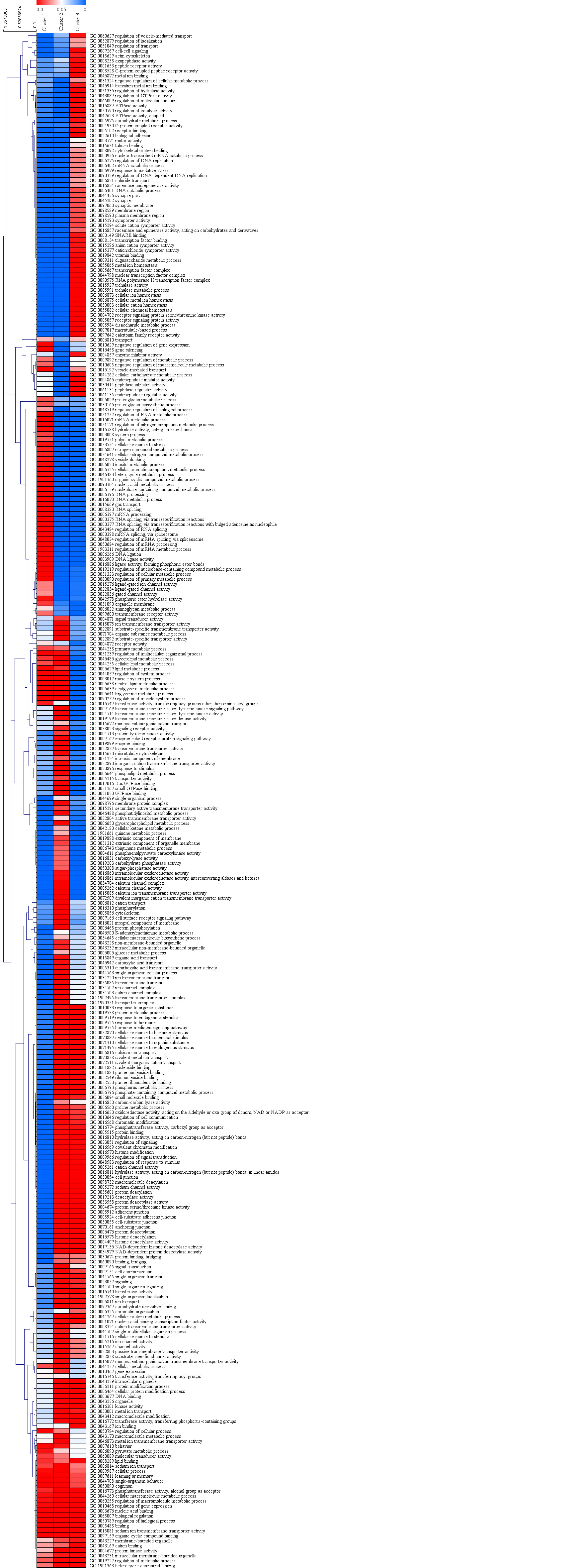

Supplement: Supplementary file 3 [file Image1.JPEG]
